# Tools for assessing the scalability of innovations in health: a systematic review

**DOI:** 10.1186/s12961-022-00830-5

**Published:** 2022-03-24

**Authors:** Ali Ben Charif, Hervé Tchala Vignon Zomahoun, Amédé Gogovor, Mamane Abdoulaye Samri, José Massougbodji, Luke Wolfenden, Jenny Ploeg, Merrick Zwarenstein, Andrew J. Milat, Nathalie Rheault, Youssoufa M. Ousseine, Jennifer Salerno, Maureen Markle-Reid, France Légaré

**Affiliations:** 1 CubecXpert, Quebec City, QC Canada; 2 Department of Social and Preventive Medicine, Université Laval, Quebec City, QC Canada; 3 Faculty of Medicine and Health Science, School of Physical and Occupational Therapy, McGill University, Montreal, QC Canada; 4 Institut national d’excellence en santé et en services sociaux (INESSS), Quebec City, QC Canada; 5 VITAM – Centre de recherche en santé durable, Université Laval, Quebec City, QC Canada; 6 Tier 1 Canada Research Chair in Shared Decision Making and Knowledge Translation, Université Laval, Quebec City, QC Canada; 7 Department of Family Medicine and Emergency Medicine, Université Laval, Quebec City, QC Canada; 8 Unité de soutien SSA Québec, Université Laval, Quebec City, QC Canada; 9 Institut national de santé publique du Québec (INSPQ), Quebec City, QC Canada; 10 School of Medicine and Public Health, University of Newcastle, Callaghan, NSW Australia; 11 Hunter Medical Research Institute, New Lambton Heights, NSW Australia; 12 Hunter New England Population Health, Wallsend, NSW Australia; 13 Aging, Community and Health Research Unit, School of Nursing, Faculty of Health Sciences, McMaster University, Hamilton, ON Canada; 14 Department of Family Medicine, Centre for Studies in Family Medicine, Schulich School of Medicine and Dentistry, Western University, London, ON Canada; 15 School of Public Health, University of Sydney, Sydney, NSW Australia; 16 Centre for Epidemiology and Evidence, NSW Ministry of Health, Sydney, Australia; 17 Santé publique France, Paris, France; 18 Canada Research Chair in Person Centred Interventions for Older Adults with Multimorbidity and their Caregivers, McMaster University, Hamilton, ON Canada; 19 Population Health and Practice-Changing Research Group, CHU de Québec Research Centre, Quebec City, QC Canada

**Keywords:** Scalability, Scaling up, Scaling, Spread, Health innovations, Patient and public involvement, Scalability assessment tool, Systematic review

## Abstract

**Background:**

The last decade has seen growing interest in scaling up of innovations to strengthen healthcare systems. However, the lack of appropriate methods for determining their potential for scale-up is an unfortunate global handicap. Thus, we aimed to review tools proposed for assessing the scalability of innovations in health.

**Methods:**

We conducted a systematic review following the COSMIN methodology. We included any empirical research which aimed to investigate the creation, validation or interpretability of a scalability assessment tool in health. We searched Embase, MEDLINE, CINAHL, Web of Science, PsycINFO, Cochrane Library and ERIC from their inception to 20 March 2019. We also searched relevant websites, screened the reference lists of relevant reports and consulted experts in the field. Two reviewers independently selected and extracted eligible reports and assessed the methodological quality of tools. We summarized data using a narrative approach involving thematic syntheses and descriptive statistics.

**Results:**

We identified 31 reports describing 21 tools. Types of tools included criteria (47.6%), scales (33.3%) and checklists (19.0%). Most tools were published from 2010 onwards (90.5%), in open-access sources (85.7%) and funded by governmental or nongovernmental organizations (76.2%). All tools were in English; four were translated into French or Spanish (19.0%). Tool creation involved single (23.8%) or multiple (19.0%) types of stakeholders, or stakeholder involvement was not reported (57.1%). No studies reported involving patients or the public, or reported the sex of tool creators. Tools were created for use in high-income countries (28.6%), low- or middle-income countries (19.0%), or both (9.5%), or for transferring innovations from low- or middle-income countries to high-income countries (4.8%). Healthcare levels included public or population health (47.6%), primary healthcare (33.3%) and home care (4.8%). Most tools provided limited information on content validity (85.7%), and none reported on other measurement properties. The methodological quality of tools was deemed inadequate (61.9%) or doubtful (38.1%).

**Conclusions:**

We inventoried tools for assessing the scalability of innovations in health. Existing tools are as yet of limited utility for assessing scalability in health. More work needs to be done to establish key psychometric properties of these tools.

*Trial registration* We registered this review with PROSPERO (identifier: CRD42019107095)

**Supplementary Information:**

The online version contains supplementary material available at 10.1186/s12961-022-00830-5.

## Background

Various innovations have been developed and successfully piloted to strengthen healthcare systems in low-, middle- or high-income countries [1–3]. A health innovation refers to a set of behaviours, routines and ways of working that are perceived as new; that aim to improve health outcomes, administrative efficiency, cost-effectiveness or user experience; and that are implemented through planned action [4–6]. But there is a global delivery gap between innovations for which evidence of effectiveness has been established and those that actually reach the people who could benefit [7, 8]. Thus, the last decade has seen growing interest in the scaling up of health innovations. Scaling up, or expanding the impact and reach of effective innovations, could reduce waste and inequalities in health settings and improve outcomes [7–9]. For example, up to 85% of all maternal, neonatal and child deaths in low- or middle-income countries could potentially be averted through scaling up of successfully piloted innovations [10]. The science of knowledge mobilization, or moving knowledge into action (also known variously as knowledge translation and implementation science), can be a key instrument for closing this gap by taking evidence-based innovations and testing strategies to move them into wider practice [11–13]. Thus, there is a need for tools to help identify evidence-based innovations that could be successfully expanded or scaled up to reach more patients in healthcare systems.

There are various definitions of scaling up [14], ranging from an increase in the number of beneficiaries, organizations or geographic sites, to more complex definitions in which expanding the variety, equity and sustainability of an innovation is also considered [1, 6, 15]. Some innovations are implemented at scale before ever going through a pilot trial or small-scale introduction [16]. This was the case with the coronavirus disease 2019 (COVID-19) vaccines in Canada, for example, which were developed elsewhere through clinical research and then introduced simultaneously nationwide at the local level. In some situations, scale-up is transnational; for example, innovations adopted first in a low- or middle-income country are then transferred or scaled up to a high-income country [17, 18]. Scale-up can be nonlinear, and is inherently complex and often political [19]. Scalability is defined as the “ability of a health innovation shown to be efficacious on a small scale and/or under controlled conditions to be expanded under real-world conditions to reach a greater proportion of the eligible population, while retaining effectiveness” [20]. Here, we consider scalability broadly as also including assessing whether the innovation can be replicated, transferred or sustained [6, 21].

Among other considerations in preparing for scale-up, decision-makers need to assess the more technical scalability components of an innovation [2, 3]. In 2003, Everett Rogers identified key innovation characteristics relevant for assessing scalability: relative advantage (which includes effectiveness), compatibility, complexity, comprehensibility (to the user), trialability, observability and potential re-invention (i.e. adaptation) [5]. Since then, others have adapted and added to these characteristics [6]. Milat’s scalability assessment tool [22], for example, based on existing frameworks, guides and checklists, is a recent and comprehensive effort to select and summarize essential components of a scale-up preparedness plan [1, 15, 21, 23]. In spite of these advances, however, scalability assessments are still often overlooked by those responsible for developing and delivering innovations in health [1, 16].

Thus, scalability assessments target certain key components or properties that are critical for scale-up. For example, many health innovations are scaled up in the absence of evidence of beneficial impact [16], a scalability component that is an essential predictor of successful scale-up [3, 6, 21]. Scalability assessments should also anticipate known pitfalls of scale-up, that is, elements that have compromised the success of scaling up, such as the replicating of harms at scale [24]. While few studies focus on scale-up failures, studies that do so can throw into relief gaps that otherwise might be overlooked [25]. Failing to involve patients and the public, especially those who may be socially excluded owing to age, ethnicity, or sex and gender, may also result in poor programmatic outcomes, as scale-up could overlook the concerns of its intended beneficiaries [14, 15, 26].

In addition to the complex strategic, political and environmental considerations surrounding scale-up, end-users (e.g. policy-makers, implementers) lack theoretical, conceptual and practical tools for guiding scalability assessments in health settings [27]. In Canada, many innovation teams have expressed the need for a validated tool for scalability assessment in primary healthcare [2, 3]. No previous knowledge synthesis has been conducted on the measurement properties (i.e. quality aspects such as reliability, validity and responsiveness) of scalability assessment tools. Thus, we aimed to review existing tools for assessing scalability of health innovations, describing how the tools were created and validated, and describing the scalability components they target. Our research question was as follows: “What tools are available for assessing the scalability of innovations in health, how were they created, what are their measurement properties, and what components do they target?”

## Methods

### Design

We performed a systematic review with a comprehensive overview of the components targeted by scalability assessment tools and their measurement properties. We adapted and followed the COnsensus-based Standards for the selection of health Measurement INstruments (COSMIN) methodology for systematic reviews [28]. We reported the review according to the Preferred Reporting Items for Systematic Reviews and Meta-Analyses (PRISMA) 2020 guidelines [29] and the COSMIN reporting recommendations [28]. In this manuscript, the noun “report” refers to a document (paper or electronic) supplying information about a study, and the noun “record” refers to the title or abstract of a report indexed in a database or website [29]. We registered this review in the International Prospective Register of Systematic Reviews (PROSPERO) on 2 May 2019 (registration identifier: CRD42019107095) [30].

### Eligibility criteria

Following the COSMIN approach, we used the following eligibility criteria.

- Construct: We included any tool aiming to assess or measure scalability of innovations in health. WHO defines health as “a state of complete physical, mental and social well-being and not merely the absence of disease or infirmity”. According to the International Classification of Health Interventions, types of health innovations could include management, prevention, therapeutic, diagnostic, other (i.e. not classified elsewhere) or unspecified [3, 31].
- Population: We included any type of stakeholder or end-user. Stakeholders refer to persons who were involved in the conception, creation or validation of the tool [32]. End-users refer to individuals such as policy-makers who are likely to use the tool to make decisions about scaling up an innovation [33]. Stakeholders can also be end-users, and both can include patients and the public, healthcare providers, policy-makers, investigators, trainees and funders [14]. End-users can be involved in the creation or validation process of the tool, and the level of their involvement may vary from minimal (i.e. receiving information about it, but with no contributing role) to coproducing the tool (i.e. participating as an equal member of the research team) [14, 34, 35].
- Instrument: We included any tool containing items proposed for assessing the scalability of an innovation in health. A tool refers to a structured instrument such as a guide, framework, questionnaire, factors, facilitators or barriers. Items refer to individual elements of the tool such as questions or statements that were mapped to targeted components.
- Measurement properties: We included any reports presenting (1) creation of a scalability assessment tool, (2) assessment of one or more measurement properties of the tool or (3) assessment of the interpretability of the tool. A measurement property is defined as a quality aspect of a tool, i.e. reliability, validity and responsiveness [28]. We included any of the following nine measurement properties: content validity, structural validity, internal consistency, cross-cultural validity or measurement invariance, reliability, measurement error, criterion validity, hypotheses testing for construct validity, and responsiveness. We excluded any study protocol and any editorial material, defined as an article that gives the opinions of a person, group or organization (e.g. editorials, commentaries and letters).

In other words, we included any empirical research which aimed to investigate the creation, validation or interpretability of a scalability assessment tool in health settings (Table 1).

**Table 1:** Criteria for considering records or reports for this review

Criteria	Inclusion	Exclusion	Question related to the criteria
Type of report	Original paper Research report Evaluation report Knowledge synthesis Government document	Editorial Commentary Opinion letter Protocol	*Is this empirical research using quantitative or qualitative methods?*
Aim of study	Development of a tool (e.g. a guide, framework, questionnaire, factors, facilitators or barriers) Assessment of one or more measurement (or psychometric) properties of a tool Assessment of the interpretability of a tool	The study did **not** present or describe a guide, framework, questionnaire, factors, facilitators or barriers (hereafter referred to as “tool”)	*Does the study present the development, validation or interpretability of a tool?*
Aim of the tool	Tool aiming to assess the scalability of an innovation (i.e. potential or readiness for scale-up, for spread, for transfer, for diffusion or for system wide implementation)	The tool is **not** intended to be used for assessing the scalability of an innovation	*Does the tool aim to evaluate the scalability of an innovation?*
Setting	Any health context	The tool is **not** intended to be used for an innovation in the field of health	*Is the tool intended to be used for innovation in the field of health?*

### Literature search

Overall, we performed a comprehensive search to identify records through both electronic databases of peer-reviewed literature and secondary searches, including hand searching relevant websites, screening reference lists of included or relevant reports, and consulting experts in the field of scale-up. There was no restriction regarding language, date or country of publication, or type of reports.

*First*, we searched Embase via embase.com, MEDLINE via Ovid, CINAHL via EBSCO, Web of Science, PsycINFO via Ovid, the Cochrane Library, and ERIC via EBSCO from their inception to 20 March 2019. An information specialist with the Unité de soutien SSA Québec [36] (NR) drafted the preliminary version of the search strategy for Ovid MEDLINE. The search terms were based on previous works to reflect three concepts: scalability [1], tool [37] and health [38]. The preliminary search strategy was reviewed by eight international experts (ABC, HTVZ, LW, JP, MZ, AJM, JS and MMR), and then by a second information specialist in the Faculty of Medicine at Université Laval (F. Bergeron) using the Peer Review of Electronic Search Strategies (PRESS) guideline [39]. The experts were university-based investigators (from Benin, Togo, Comoros, Australia, and Canada) and experts in knowledge mobilization, health services research, health research methodology and scaling up. We resolved any disagreements through a consensus meeting between the two information specialists and a third party (ABC and HTVZ). The search terms were adapted to the above-mentioned databases by removing search terms related to the concept of health in all biomedical databases—the difference in the number of records found in MEDLINE when removing health-related terms was minimal (104 records out of a total of 2528). Details of the search strategy in each electronic database can be found in the appendix (Additional file 1).

*Second*, we identified other records by searching relevant websites, screening reference lists of included or relevant reports, and consulting experts in the field of scale-up. This approach is promoted as a way of reducing publication bias [40]. We consulted Google Scholar, Google web search, and the websites of a list of 24 Canadian and international organizations in both English and French from 10 October to 20 December 2019 (Additional file 2). In French, we used the following keywords: “potentiel de mise à l’échelle”, “potentiel de passage à grande échelle”, “transférabilité”, “mise à l’échelle”, “passage à grande échelle”, “accroissement d’échelle”, “passage à l’échelle” and “diffusion”. In English, we used terms related to the concept of scalability including scalability, transferability, readiness, scale, scaling, upscaling, up-scaling, and spread (Additional file 1). We also established a list of experts in the field of scale-up and asked them via email about documentation of tools they had created or knew about, from 5 to 29 May 2020 (Additional file 3). The list of experts included authors of reports included in this systematic review, authors of reports included in our previous systematic review [1], members of the 12 Canadian Institutes of Health Research (CIHR)-funded Community-Based Primary Health Care (CBPHC) teams [2, 41], and members of the Research on Patient-Oriented Scaling-up (RePOS) network [14].

### Selection process

*First*, we operationalized eligibility criteria using questions with the following responses: “met”, “not met” and “unclear”. Five author reviewers (ABC, AG, MAS, JM and YMO) independently screened a random sample of 5% of records identified with our literature search. We discussed the results of this pilot and reviewed the eligibility criteria. *Second*, two senior end-users and experts in scaling up (JP and MZ) independently screened five records and suggested a minor change in wording to clarify eligibility criteria. *Third*, the same five reviewers independently piloted the selection of another random sample of 5% of the remaining records. We calculated inter-reviewer agreement between these five reviewers using the weighted Cohen’s kappa [42] and considered it substantial when we reached a value of at least 0.60 [43]. *Fourth*, the five reviewers (ABC, AG, MAS, JM and YMO) independently screened all remaining records. We detail the records assignment and kappa calculation in the appendix (Additional file 4). *Fifth*, two reviewers (ABC and MAS) assessed all potentially relevant reports to identify reports meeting the eligibility criteria. For all ineligible reports, we documented the main reason for exclusion. *Finally*, in all steps, we resolved all disagreements through consensus among reviewers in face-to-face meetings and, when required, with the project leader (ABC). Records that referred to the same report were considered duplicates, but records that referred to reports that were merely similar were considered unique [29]. We used EndNote X9 software to identify duplicates and an Excel form for the selection process.

### Data collection process

We developed an Excel form to guide extraction of variables based on the COSMIN manual [28]. Six reviewers (ABC, HTVZ, AG, MAS, JM and YMO) performed a calibration exercise to ensure that the form captured all relevant data. Then two reviewers (ABC and MAS) independently extracted data using the Excel form. The following information was extracted from each included unique report:

- characteristics of included tools (e.g., type, date of issue or publication, funding support, language, stakeholder, open-access source, name, scalability components targeted, content and pitfall predictions);
- intended context of use (e.g. income level of country, healthcare level, focus area, end-user and aim); and
- data that could be considered sources of validity for measurement properties. For example, data regarding the tool’s content validity could include test blueprint, representativeness of items in relation to the scalability component, logical or empirical relationship of content tested to scalability component, strategies to ensure appropriate content representation, item writer qualifications, and analyses by experts regarding how adequately items represent the content of the scalability component [44].

All disagreements were resolved through consensus between ABC and MAS in face-to-face and virtual meetings. We used Microsoft Teams for the virtual meetings.

### Quality assessment of tools

We used the COSMIN Risk of Bias checklist to assess the methodological quality of included tools [28]. This checklist contains one box with standards for assessing the tool’s methodological quality and nine boxes for assessing the methodological quality of studies that reported measurement properties for tools. In this review, because there were very limited data on content validity and no data on other measurement properties, we assessed the methodological quality of tool creation only, which is also part of the content validity. Two reviewers (ABC and MAS) independently assessed the quality of all included tools after a pilot using a sample of two tools. We resolved all disagreements through consensus between ABC and MAS in virtual meetings using Microsoft Teams.

The COSMIN standards for tool creation consist of 35 items divided into two parts [45]: Part A addresses the quality of the design and Part B the quality of the pilot study. Part A includes a concept elicitation study performed with end-users to identify relevant items for a new tool, and a clear description of the construct and how it relates to the theory or conceptual framework from which it originates. Part B includes a pilot study performed with end-users to evaluate comprehensiveness and comprehensibility. Each standard is scored on a four-point rating scale: “very good”, “adequate”, “doubtful” or “inadequate”. A standard is rated as “doubtful” if it is doubtful whether the quality aspect is adequate (i.e. minor methodological flaws), and “inadequate” when evidence is provided that the quality aspect is not adequate (i.e. important methodological flaws) [28]. Where a score for a standard was not requested, the option “not applicable” was available. Total scores are determined separately for concept elicitation and pilot test. A total score per tool is obtained by taking the lowest rating of any item (i.e. worst score counts).

### Data analysis

We analysed and summarized extracted data using a narrative approach involving framework and content analysis [46]. We created an integrated framework of categories for the purpose of this study based on recent work on scaling up. All classification was carried out independently by two reviewers (ABC and MAS) and all disagreements were resolved through consensus in virtual meetings using Microsoft Teams. We used the PRISMA 2020 flowchart to describe the process of tool selection [29]. We summarized the main characteristics of tools, including components targeted by the tools and their methodological quality, in a tabular display using SAS 9.4 software.

*First*, we classified each tool using the three types: (1) scale, (2) checklist or (3) set of criteria. To be considered a scale, each item within the tool had to have a numeric score attached to it so that an overall summary score could be calculated. To be considered a checklist, the tool had to include multiple items to observe for scalability criteria to be met. To be considered “criteria”, the tool had to include a list of items (questions or statements) with no proposed responses. *Second*, we mapped each item of each tool to the following 12 possible components targeted by the tool: (${\text{C}}_{1}$) health problem addressed by the innovation; (${\text{C}}_{2}$) development process of the innovation; (${\text{C}}_{3}$) innovation characteristics; (${\text{C}}_{4}$) strategic, political or environmental context of the innovation; (${\text{C}}_{5}$) evidence available for effectiveness of the innovation; (${\text{C}}_{6}$) innovation costs and quantifiable benefits; (${\text{C}}_{7}$) potential for implementation fidelity and adaptation of the innovation; (${\text{C}}_{8}$) potential reach and acceptability to the target population; (${\text{C}}_{9}$) delivery setting and workforce; (${\text{C}}_{10}$) implementation infrastructure required for scale-up; (${\text{C}}_{11}$) sustainability (i.e. longer-term outcomes of the scale-up); and (${\text{C}}_{{{\text{Other}}}}$) other components. This classification was based on Milat’s 10-component framework [22], to which we added items related to the development process of the innovation such as the use of a theoretical, conceptual or practical framework (${\text{C}}_{2}$) [2, 3], which is the primary stage of scale-up [16]. *Third*, we determined whether each tool included items related to eight potential pitfalls to be anticipated when planning scale-up of the innovation. Six of those pitfalls were based on a rapid review of points of concern regarding the success or failure of scale-up efforts [24]. To these six pitfalls we added patient and public involvement and sex and gender. These were demonstrations that development or piloting of the innovation had not excluded its targeted beneficiaries (e.g. excluding women in a programme about women’s health) [1, 14, 15, 26]. The expanded pitfalls thus consisted of the following: (${\text{P}}_{1}$) sex and gender considerations; (${\text{P}}_{2}$) patient and public involvement﻿; (${\text{P}}_{3}$) the difficulty of cost-effectiveness estimates﻿; (${\text{P}}_{4}$) the production of health inequities﻿; (${\text{P}}_{5}$) scaled-up harm﻿; (${\text{P}}_{6}$) ethics (e.g. informed consent at scale)﻿; (${\text{P}}_{7}$) top-down approaches (i.e. the needs, preferences and culture of beneficiaries of the innovation may be forgotten when scale-up is directed from above)﻿; and (${\text{P}}_{8}$) context (e.g. difficulty in adapting the innovation to certain contexts). *Finally*, we adopted a previous rating system to quantify the extent to which sources of validity evidence for measurement properties of the tools were reported: 0 = “no discussion or data presented as a source of validity evidence”; 1 = “data that weakly support the validity evidence”; 2 = “some data (intermediate level) that support the validity evidence, but with gaps”; and 3 = “multiple sets of data that strongly support the validity evidence” [44].

## Results

### Study selection

**Fig. 1:**
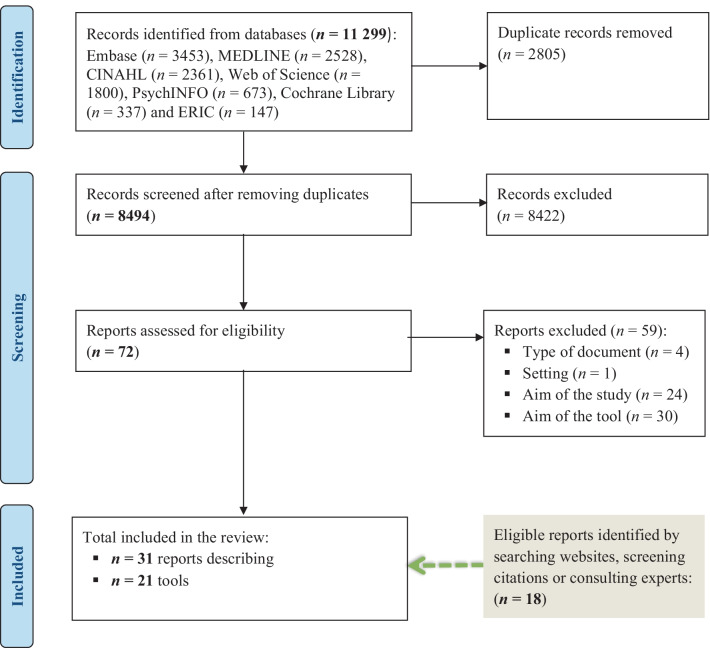
PRISMA 2020 flow diagram of the tool inclusion process

Our electronic search identified 11 299 potentially relevant records. Of these, 2805 were duplicates, leaving 8494 records. Of these, 8422 did not meet the review criteria. With the second random sample of 5% of the 8494 records, we found substantial pair inter-reviewer agreements for decisions regarding inclusion, with kappa values ranging from 0.66 to 0.89 across all reviewers (Additional file 4). Finally, we reviewed a total of 72 reports, retained 13 [2, 47–58] and excluded 59 [59–117] (Additional file 5). In addition, our secondary searches led to the inclusion of 18 additional reports [3, 6, 20–22, 118–130]. Overall, we included a total of 31 reports from all sources [2, 3, 6, 20–22, 47–58, 118–130], which described a total of 21 unique tools (Fig. 1). We included the following tools: the Innovation Scalability Self-administered Questionnaire (ISSaQ) [2, 3], the AnalySe de la Transférabilité et accompagnement à l’Adaptation des Interventions en pRomotion de la santE (ASTAIRE) [53, 54], the Process model for the assessment of transferability (PIET-T) [55], the CORRECT attributes [6, 121, 122], the scalability assessment framework [57], the Intervention Scalability Assessment Tool (ISAT) [22], the Readiness to Spread Assessment Scoring Sheet [125], the Readiness to Receive Assessment Scoring Sheet [126], the Applicability and Transferability of Evidence Tool (A&T Tool) [118, 119], the Scalability Assessment and Planning (SAP) Toolkit [130] and the Scalability Checklist [127–129]. We did not find names for 10 of the tools [20, 21, 47–52, 56, 58, 120, 123, 124].

### Characteristics of tools

Characteristics of included tools are outlined in Table 2.

**Table 2:** Characteristics of included tools

Name (abbreviation)^a^ [References]	Type and source						Scalability component targeted by tools^b^											Number of items	Pitfall predictions^c^
	Type	Year of issue or publication	Source of funding	Language	Type of stakeholder	Open-access source	C_1_	C_2_	C_3_	C_4_	C_5_	C_6_	C_7_	C_8_	C_9_	C_10_	C_11_		
Innovation Scalability Self-administered Questionnaire (ISSaQ) [2, 3]	Checklist	2017	Governmental organization	English, French	Researcher	Peer-reviewed journal		✓		✓	✓	✓	✓	✓	✓	✓	✓	16	${\text{P}}_{3,8}$
AnalySe de la Transférabilité et accompagnement à l’Adaptation des Interventions en pRomotion de la santE (ASTAIRE) [53, 54]		2013	Governmental organization	English, French	Researcher	Peer-reviewed journal	✓	✓		✓	✓	✓	✓	✓	✓	✓		23	${\text{P}}_{2,3,7,8}$
WHO/ExpendNet [123, 124]		2011	Governmental and nongovernmental organizations	English	Not found	Organizational website	✓	✓	✓	✓	✓	✓			✓	✓		23	${\text{P}}_{1,3,4,7,8}$
Scalability Checklist [127–129]		2016	Nongovernmental organization	English	Not found	Organizational website	✓			✓	✓	✓	✓	✓	✓	✓		7	${\text{P}}_{3,8}$
Baker et al. [47]	Criteria	2011	Governmental organization	English	Not found	ResearchGate			✓					✓	✓	✓		16	${\text{P}}_{1,2,6,7,8}$
Bennett et al. [48]		2017	Governmental organization	English	Not found	Peer-reviewed journal			✓		✓	✓	✓	✓		✓	✓	8	${\text{P}}_{3,8}$
Burchett et al. [50]		2011	Not found	English	Researcher	ResearchGate			✓		✓	✓	✓	✓	✓		✓	17	${\text{P}}_{3,5,8}$
Burchett et al. [51]		2012	Governmental organization	English	Clinician, policy-maker, researcher	Not found	✓		✓		✓	✓	✓	✓	✓		✓	15	${\text{P}}_{3,6,7,8}$
Cambon et al. [52]		2012	Governmental organization	English	Not found	Peer-reviewed journal	✓	✓	✓	✓		✓	✓	✓	✓	✓		32	${\text{P}}_{1,3,6,7,8}$
Process model for the assessment of transferability (PIET-T) [55]		2018	Governmental organization	English	Not found	Peer-reviewed journal	✓	✓	✓	✓	✓	✓	✓	✓	✓	✓	✓	14	${\text{P}}_{2,8}$
Spicer et al. [56]		2014	Nongovernmental organization	English	Policy-maker, researcher, civil society organizations	Peer-reviewed journal	✓		✓	✓	✓	✓	✓	✓	✓			22	${\text{P}}_{3,8}$
Wang et al. [58]		2005	Not found	English	Not found	Not found	✓			✓			✓	✓	✓	✓		12	${\text{P}}_{6,8}$
Milat et al. [20, 21, 120]		2012	Governmental organization	English	Policy-maker, researcher	Peer-reviewed journal, ResearchGate, organizational website				✓	✓	✓	✓	✓	✓	✓	✓	21	${\text{P}}_{3,5,8}$
CORRECT attributes^d^ [6, 121, 122]		2010	Governmental organization	English, French, Spanish	Not found	Organizational website	✓		✓		✓	✓	✓		✓	✓		17	${\text{P}}_{3,6,8}$
Bhattacharyya et al. [49]	Scale	2017	Governmental and nongovernmental organizations	English	Policy-maker	Peer-reviewed journal			✓	✓	✓		✓		✓			8	${\text{P}}_{2,3,8}$
Scalability assessment framework [57]		2018	Nongovernmental organization	English	Not found	Not found	✓	✓	✓	✓		✓	✓		✓	✓	✓	16	${\text{P}}_{3,7,8}$
Intervention Scalability Assessment Tool (ISAT) [22]		2019	Governmental organization	English	Clinician, policy-maker, researcher	Organizational website	✓		✓	✓	✓	✓	✓	✓	✓	✓	✓	19	${\text{P}}_{3,8}$
Readiness to Spread Assessment Scoring Sheet [125]		2013	Not found	English	Not found	Organizational website			✓		✓		✓	✓		✓	✓	4	${\text{P}}_{5}$
Readiness to Receive Assessment Scoring Sheet [126]		2013	Not found	English	Not found	Organizational website				✓					✓	✓		4	${\text{P}}_{8}$
Applicability and Transferability of Evidence Tool (A&T Tool) [118, 119]		2007	Governmental organization	English, French	Not found	Organizational website	✓			✓		✓	✓	✓	✓			21	${\text{P}}_{3,7,8}$
Scalability Assessment and Planning (SAP) Toolkit [130]		2018	Not found	English	Researcher	Organizational website					✓		✓			✓	✓	5	Not found

^a^We did not find names for 10 of the tools, in which case we indicated names of authors or organizations

^b^Scalability components targeted: (${\text{C}}_{1}$) health problem addressed by the innovation; (${\text{C}}_{2}$) development process of the innovation; (${\text{C}}_{3}$) innovation characteristics; (${\text{C}}_{4}$) strategic, political or environmental context of the innovation; (${\text{C}}_{5}$) evidence available for effectiveness of the innovation; (${\text{C}}_{6}$) innovation costs and quantifiable benefits; (${\text{C}}_{7}$) potential for implementation fidelity and adaptation of the innovation; (${\text{C}}_{8}$) potential reach and acceptability to the target population; (${\text{C}}_{9}$) delivery setting and workforce; ${\text{C}}_{10}$) implementation infrastructure required for scale-up; and (${\text{C}}_{11}$) sustainability (i.e. longer-term outcomes of the scale-up)

^c^Pitfalls of scale-up relate to: (${\text{P}}_{1}$) sex and gender considerations; (${\text{P}}_{2}$) patient and public involvement; (${\text{P}}_{3}$) the difficulty of cost-effectiveness estimates; (${\text{P}}_{4}$) the production of health inequities; (${\text{P}}_{5}$) scaled-up harm; (${\text{P}}_{6}$) ethics (e.g. informed consent at scale); (${\text{P}}_{7}$) top-down approaches (i.e. the needs, preferences and culture of beneficiaries of the innovation may be forgotten when scale-up is directed from above); and (${\text{P}}_{8}$) context (e.g. difficulty in adapting the innovation to certain contexts)

^d^CORRECT attributes: C—credible in that they are based on sound evidence or advocated by respected persons or institutions; O—observable to ensure that potential users can see the results in practice; R—relevant for addressing persistent or sharply felt problems; R—relative advantage over existing practices so that potential users are convinced the costs of implementation are warranted by the benefits; E—easy to install and understand rather than complex and complicated; C—compatible with the potential users’ established values, norms and facilities; fit well into the practices of the national programme; and T—testable so that potential users can see the innovation on a small scale prior to large-scale adoption

✓ is a checkmark for the item

Type and source of tools: most tools were criteria (*n* = 10, 47.6%), followed by scales (*n* = 7, 33.3%) and checklists (*n* = 4, 19.0%). Included tools were created or published from 2005 onwards and the majority since 2010 (*n* = 19, 90.5%). Their creation was funded by governmental or nongovernmental organizations (*n* = 16, 76.2%). All tools were in English; three were translated into French only (14.3%) and one into French and Spanish (4.8%). Most tools were available through open-access peer-reviewed journals, ResearchGate or organizational websites (*n* = 18, 85.7%).

Scalability components: all tools targeted multiple components. The most frequently targeted components were potential implementation fidelity and adaptation (81.0%), delivery setting and workforce (81.0%), and implementation infrastructure (81.0%). The three least frequently targeted were health problems addressed by the innovation (57.1%), sustainability (47.6%), and development process of innovations (28.6%) (Table 2).

Content of tools: tools contained a total of 320 items (e.g. questions, statements) mapping to targeted components (Additional file 6). There was a median of 16 items per tool (interquartile range: 13 items). In 286 items, just one scalability component was targeted; in 27 items, two scalability components were targeted; in five items, three scalability components were targeted; and in two items, four scalability components were targeted. Most items covered delivery setting and workforce (68 items), reach and acceptability for the target population (62 items), and evidence available for effectiveness of the innovation (42 items). Components least covered by items were problem addressed by the innovation (19 items), development process of the innovation (16 items), and sustainability (12 items).

Pitfall predictions: most tools included items that considered contextual pitfalls (90.5%) and cost-effectiveness estimation pitfalls (71.4%). Pitfalls least considered were scaled-up harms (14.3%) and health inequities (4.8%) (Table 2).

Stakeholder involvement: no information on stakeholder involvement in tool creation or validation was found for 12 out of the 21 tools (57.1%) (Table 2). No studies reported involving patients or the public, for example, or reported on the sex of tool creators. Tool creation involved single (*n *= 5, 23.8%) or multiple (*n *= 4, 19.0%) types of stakeholders, including clinicians, policy-makers, researchers and civil society organizations (Table 2).

### Intended context of use

Eight tools did not report the income levels of countries for which they were created (38.1%) (Table 3). Six tools were reported as created for use in high-income countries (28.6%), four in low- or middle-income countries (19.0%), two in both (9.5%), and one for transnational transfers from low- or middle-income to high-income contexts (4.8%).

**Table 3:** Characteristics of intended context of use of included tools

Name (abbreviation)^a^ [References]	Income level context	Healthcare level	Focus area	Sex or gender of beneficiaries of the targeted innovations	End-user of tool	Aim of tool	Degree of report of validity evidence for content validity^b^	Methodological quality of tools^c^
Innovation Scalability Self-administered Questionnaire (ISSaQ) [2, 3]	High-income country	Primary healthcare	Not found	Female, male	Clinician, policy-maker, researcher	Assess the scalability of innovations in primary healthcare	2	Inadequate
AnalySe de la Transférabilité et accompagnement à l’Adaptation des Interventions en pRomotion de la santE (ASTAIRE) [53, 54]	High-income country	Not found	Health prevention or promotion	Not found	Not found	Assess transferability and adaptation of health promotion innovations	3	Doubtful
WHO/ExpendNet [123, 124]	Not found	Not found	Not found	Not found	Researcher, policy-maker, programme manager, funder	Assess the scalability of programmatic research; provide a quick assessment of how easy or difficult it will be to scale up a project that is being planned or proposed or is in the process of implementation	2	Doubtful
Scalability Checklist [127–129]	Not found	Primary healthcare	Reproductive, maternal, newborn, child or adolescent health	Not found	Not found	Prioritize alternatives and identify actions that can be taken to simplify the scaling-up process	2	Doubtful
Baker et al. [47]	Not found	Primary healthcare, home care, public or population health	Reproductive, maternal, newborn, child or adolescent health	Female	Not found	Assess the applicability and transferability of innovations to the Aboriginal and Torres Strait Islander setting	2	Inadequate
Bennett et al. [48]	Low- or middle-income country	Primary healthcare	Reproductive, maternal, newborn, child or adolescent health	Not found	Not found	Explore feasibility and effectiveness of health innovations	1	Inadequate
Burchett et al. [50]	Not found	Public or population health	Not found	Not found	Not found	Assist in the assessment of applicability and transferability	2	Inadequate
Burchett et al. [51]	Low- or middle-income country	Primary healthcare, public or population health	Reproductive, maternal, newborn, child or adolescent health	Not found	Not found	Assess a study’s applicability and transferability	2	Inadequate
Cambon et al. [52]	Low-, middle- or high-income country	Public or population health	Health prevention or promotion	Not found	Not found	Guide and assess transferability	2	Inadequate
Process model for the assessment of transferability (PIET-T) [55]	Not found	Not found	Health prevention or promotion	Not found	Not found	Accompany the steps for determining transferability	2	Inadequate
Spicer et al. [56]	Low- or middle-income country	Primary healthcare	Reproductive, maternal, newborn, child or adolescent health	Not found	Researcher	Increase the prospects of government adoption and community uptake of innovations at scale	1	Inadequate
Wang et al. [58]	Not found	Public or population health	Not found	Not found	Not found	Assess applicability and transferability from a study setting to a local setting using evidence about both the local setting and the public health innovation of interest	1	Inadequate
Milat et al. [20, 21, 120]	High-income country	Public or population health	Health prevention or promotion	Not found	Policy-maker, researcher	Explore whether an innovation is scalable	2	Inadequate
CORRECT attributes^d^ [6, 121, 122]	Low- or middle-income country	Primary healthcare, public or population health	Reproductive, maternal, newborn, child or adolescent health	Not found	Researcher, manager, funder	Assess the attributes that determine the scalability of the innovation and identify needed actions	2	Doubtful
Bhattacharyya et al. [49]	Transnational	Not found	Not found	Not found	Policy-maker, funder	Assess promising low- or middle-income country innovations for adaptation in high-income countries and identify those with high potential for more in-depth review and evaluation	3	Doubtful
Scalability assessment framework [57]	Low-, middle- or high-income country	Public or population health	Education, nutrition, sanitation, hygiene or international development more broadly	Female, male	Not found	Expand or replicate as part of a planned scaling-up process	2	Inadequate
Intervention Scalability Assessment Tool (ISAT) [22]	High-income country	Public or population health	Not found	Not found	Clinician, policy-maker	Assist practitioners, policy-makers, programme managers, and researchers to determine the scalability of a discrete health programme	3	Doubtful
Readiness to Spread Assessment Scoring Sheet [125]	High-income country	Not found	Not found	Not found	Manager	Help programme champions and leadership understand whether a promising practice is ripe for successful spread across organizations	1	Inadequate
Readiness to Receive Assessment Scoring Sheet [126]	High-income country	Not found	Not found	Not found	Manager	Help a site determine its readiness to receive an effective practice from elsewhere	1	Inadequate
Applicability and Transferability of Evidence Tool (A&T Tool) [118, 119]	Not found	Public or population health	Not found	Not found	Manager	Assist public health managers and planners in decision-making about programme priorities for their community	2	Doubtful
Scalability Assessment and Planning (SAP) Toolkit [130]	Not found	Not found	Reproductive, maternal, newborn, child or adolescent health	Not found	Not found	Guide scaling and assessment planning with corrective actions to strengthen or enable scale-up	1	Doubtful

^a^We did not find names for 10 of the tools, in which case we indicate names of authors or organizations

^b^We found no discussion or data presented as a source of validity evidence for the eight other measurement properties. 1 = Only a limited amount of data (e.g. simply listing items without justification); 2 = listing items with some references and justifications, limited description of the process for creating the tool; 3 = well-defined process for developing tool content, including both an explicit theoretical, conceptual or practical basis for the tool items and systematic item review by experts

^c^According to COSMIN definitions, a standard is rated as “doubtful” if it is doubtful whether the quality aspect is adequate (i.e. minor methodological flaws), and “inadequate” when evidence is provided that the quality aspect is not adequate (i.e. important methodological flaws)

^d^CORRECT attributes: C—credible in that they are based on sound evidence or advocated by respected persons or institutions; O—observable to ensure that potential users can see the results in practice; R—relevant for addressing persistent or sharply felt problems; R—relative advantage over existing practices so that potential users are convinced the costs of implementation are warranted by the benefits; E—easy to install and understand rather than complex and complicated; C—compatible with the potential users’ established values, norms and facilities; fit well into the practices of the national programme; and T—testable so that potential users can see the innovation on a small scale prior to large-scale adoption

Seven tools did not report which healthcare levels they were created for (33.3%) (Table 3). The largest proportion of tools for which this information was reported were created for public or population health (47.6%), primary healthcare (33.3%) or home care (4.8%) initiatives.

Nine tools did not report on the focus area (42.9%) (Table 3). The largest proportion of tools for which this information was reported were created for innovations related to reproductive, maternal, newborn, child or adolescent health (*n* = 7, 33.3%).

We found no information about intended end-users for 11 tools (52.4%) (Table 3). Tools for which this information was reported were intended for researchers, policy-makers, programme managers, healthcare providers or funders (*n* = 10, 47.6%). No tool was created for lay end-users including patients or the public.

### Measurement properties of tools

All tools presented information for content validity, but most tools (*n* = 18, 85.7%) provided limited information (e.g. simply listing items without justification, limited description of the process for creating the tool). Only three tools (14.3%) provided multiple sets of information that strongly supported content validity, such as descriptions and origins of constructs, or comprehensibility and comprehensiveness of items. No tool reported on the other measurement properties.

### Methodological quality of tools

According to COSMIN standards, the methodological quality of tools was deemed inadequate in 61.9% of cases (*n* = 13) and doubtful in 38.1% of cases (*n* = 8) (Table 3). The main reason was that design requirements were not met: for example, there was no clear description of the target population, context of use, or the tool’s evaluative or predictive purpose.

## Discussion

We reviewed tools proposed for assessing the scalability of innovations in health. Altogether, identified tools targeted 11 scalability components and predicted eight pitfalls of scale-up. All included tools were created or published since 2005, but their methodological quality was inadequate or doubtful. No studies reported that patients or the public were involved in the creation or validation process of tools, and there was limited information on how the tools were intended to be used or on their intended end-users. These findings lead us to make the following observations.

*First*, all items found in the included tools were covered by our 11 defined scalability components, confirming that these classifications come close to reflecting the full range identified by others [22], and were enriched by items contributing to avoiding identified pitfalls such as replication of harms. Scalability assessment should ensure that innovations do not replicate social inequities when implemented at scale [15, 24, 131, 132]. For example, if the design of an innovation to be scaled up was based on the male body as the norm [131], its scale-up could reproduce harmful outcomes at scale. This is the case with the conventional seat belt: Seat belts are not tested with pregnant women, and their design has undergone almost no changes since they were first patented in 1958 [133]. Yet car crashes are the main cause of foetal deaths related to maternal trauma. The forces of the seat belt against a pregnant woman’s abdomen leads to placental abruption, causing foetal death [133]. More scalability assessments should also involve patients and the public [1, 14]. For example, members of the advisory committee, together with patient representatives and other stakeholders, could visit actual or potential sites to review arrangements for the project and to assess the potential for scale-up if the innovation proves successful. Discussion with providers, programme managers and community members could provide insights into how the project will be implemented on the ground and possible challenges and opportunities for scaling up, and could inspire reflection on possible adjustments to enhance its scalability [15, 124]. Certain scalability components could be less relevant for some innovations depending on the political circumstances, or on whether they are outcome evaluations under ideal circumstances (efficacy) or real-world circumstances (effectiveness) [2, 3, 15]. In addition, epidemics (e.g. COVID-19) have highlighted how dramatically scalability considerations can change when the world changes [1, 15].

*Second*, included tools were created or published since 2005, had inadequate or doubtful methodological quality, and most were of the “criteria” type. As key psychometric properties of these tools are yet to be established, for many of the tools there is still insufficient evidence to justify their claims. Future reviews involving the use of included tools should begin at the year 2005. Our results suggest that scalability assessment tools for health are still in their infancy. Previous studies confirm this, particularly in high-income countries [1, 22, 27, 134]. Indeed, the sophistication of our included tools varied from a simple list of items (i.e. criteria) to elaborate scales [135], although none of these had been validated [22, 27]. There were also important limitations in terms of sample representativity in the creation or validation of tool content. Intended context of use, for example, and content validity, the primary measurement property, were not fully addressed in most of the included tools [135]. However, we believe that content validation may increase over time as we learn more about the notion of scalability [136]. Nevertheless, for end-users wanting to adopt an existing tool or create a new one, we propose a useful inventory of items (Additional file 6). We also hope to create a repertory of existing items whose language is accessible to lay end-users, including patients and the public. This will contribute to increasing patient and public involvement in the science and practice of scale-up in health and social services [14].

*Third*, we noticed an absence of patient and public involvement in the creation of the scalability assessment tools. Patient perspectives are not only essential in innovation development; they are also important in the creation of scalability assessment tools [14, 15], asking the right questions and providing suggestions regarding items to include [135]. Although researchers, clinicians and policy-makers may be well positioned to describe the nature, scope and impact of a health problem that is being addressed, only those who experience the issues can report on the more subjective elements [135]. When appropriate, innovation teams have a responsibility to work with target patients to anticipate potential benefits and risks associated with scaling up, and to learn what risks they are willing to accept at each step of scale-up [15]. In practice, however, involving multiple stakeholders including patients and the public in the scalability assessments is a highly complex process [14, 15]. We have established the RePOS network to build patient-oriented research capacity in the science and practice of scaling up and ensure that patients, the public and other stakeholders are meaningfully and equitably engaged [14]. This international network will undertake the next phase of this review, conducting a multi-stakeholder consensus exercise to propose patient-oriented scalability assessment tools.

*Finally*, we acknowledge that our findings should be interpreted with caution. First, the interpretability criteria for what constitutes a useful item are not met by all items listed in our inventory (e.g. reading level, lack of ambiguity, asking only a single question) [135, 137]. However, at this early stage in the creation of scalability assessment tools, our interest is in creating an item pool. We aimed to be as inclusive as possible, even to the point of being overinclusive, as nothing can be done after the fact to compensate for items we neglected to include. Indeed, our research findings can be used to detect and weed out poor items using interpretability criteria proposed in the literature for item selection (Additional file 7) [135, 137, 138]. Second, characteristics of the innovation are important in scalability assessments, but there are other important, equally relevant assessments. Examples include comparing effects over time, namely at different stages of scale-up, so that innovations can be refined as coverage expands [27], and taking into account ongoing interactions between the innovation and its potential contexts [21, 23].

## Conclusions

We reviewed and inventoried tools proposed for assessing the scalability of innovations in health and described the scalability components they targeted. Overall, the included tools covered many components of scalability and helped predict the pitfalls of scale-up in health such as the replication of harms at scale. However, our findings show that these tools are still at an early stage of creation and their key psychometric properties are yet to be established. Scalability is a new concept, and as our understanding of this construct evolves, we will often need to revise tools accordingly. Our review may aid future investigators in weighting or prioritizing where planning and actions for scale-up should focus. Future studies could further compare and contrast the identified tools to illuminate the many perspectives on scale-up and the diverse approaches needed. Further analyses of our identified tools could also deepen understanding of how implementers, including patient partners, evaluate scalability components and how tools differ in their incorporation of evidence about acceptability. We also need to identify further scalability components, nuances of components already identified, and precisely how each scalability component contributes to the scale-up process.

## Supplementary Information

**Additional file 1.** Search strategy.

**Additional file 2.** List of relevant websites used to identify potential eligible records.

**Additional file 3.** Email sent to experts to identify potential eligible records.

**Additional file 4.** Records selection process.

**Additional file 5.** List of excluded reports with reason for exclusion.

**Additional file 6.** List of identified items.

**Additional file 7.** Interpretability criteria for selecting items.

## Data Availability

Please send all requests for study data or materials to Dr Ali Ben Charif (ali.bencharif@gmail.com) or Dr France Légaré (france.legare@mfa.ulaval.ca).

## Abbreviations

ASTAIRE: AnalySe de la Transférabilité et accompagnement à l’Adaptation des Interventions en pRomotion de la santE
A&T Tool: Applicability and Transferability of Evidence Tool
CBPHC: Community-Based Primary Health Care
CIHR: Canadian Institutes of Health Research
CORRECT: C—Credible in that they are based on sound evidence or advocated by respected persons or institutions; O—observable to ensure that potential users can see the results in practice; R—relevant for addressing persistent or sharply felt problems; R—relative advantage over existing practices so that potential users are convinced the costs of implementation are warranted by the benefits; E—easy to install and understand rather than complex and complicated; C—compatible with the potential users’ established values, norms and facilities; fit well into the practices of the national programme; and T—testable so that potential users can see the innovation on a small scale prior to large-scale adoption
COSMIN: COnsensus-based Standards for the selection of health Measurement INstruments
COVID-19: Coronavirus disease 2019
ISAT: Intervention Scalability Assessment Tool
ISSaQ: Innovation Scalability Self-administered Questionnaire
PIET-T: Process model for the assessment of transferability
PRESS: Peer Review of Electronic Search Strategies
PRISMA: Preferred Reporting Items for Systematic Reviews and Meta-Analyses
PROSPERO: International Prospective Register of Systematic Reviews
RePOS: Research on Patient-Oriented Scaling-up
SAP: Scalability Assessment and Planning
