# Supplementary material for: Tools for assessing the scalability of innovations in health: a systematic review
Source: Health Res Policy Syst. 2022 Mar 24;20:34. doi: 10.1186/s12961-022-00830-5 (PMC8943495; doi:10.1186/s12961-022-00830-5)
Supplement: Supplementary file 1 — Additional file 1. Search strategy. [file 12961_2022_830_MOESM1_ESM.docx]

**Additional file 1:** Search strategy

### Ovid Medline (2019-03-20)

| **Concept** | **Keyword** | **Number** | **Result** |
| --- | --- | --- | --- |
| Tools for assessing (Controlled vocabulary) | exp guideline/ or exp Checklist/ or "Surveys and Questionnaires"/ | #1 | 456 641 |
| Tools for assessing (Free text) | ((questionnaire$ or survey$ or instrument* or tool* or score* or scale$ or guide* or criterion or checklist$ or "check list$" or framework$) adj4 (assess* or evaluat* or measur* or analy* or track* or observe or observing or observation* or predict*)).ti.  or ((questionnaire$ or survey$ or instrument* or tool* or score* or scale$ or guide* or criterion or checklist$ or framework$) adj4 (assess* or evaluat* or measur* or analy* or track* or observe or observing or observation* or predict*)).ab.  or ((questionnaire$ or survey$ or instrument* or tool* or score* or scale$ or guide* or criterion or checklist$ or framework$) adj4 (assess* or evaluat* or measur* or analy* or track* or observe or observing or observation* or predict*)).kw. | #2 | 604 033 |
| Tools for assessing | #1 or #2 | #3 | 966 924 |
| Scalability (Controlled vocabulary) | Technology Transfer/ | #4 | 2 016 |
| Scalability (Free text) | ((("scaling" or widespread or spread or "rolling out" or "roll out" or "scale$ up" or "scale$ out" or upscaling or scalability or scalable or ((bring* or brought or taking or take* or increas* or going or implement* or econom*) adj5 scal*)) adj5 (innovation$ or intervention$ or technolog* or practice* or care or initiative* or program* or product* or therap* or service*)) or "reverse innovation*" or "trickle-up innovation*" or (transfer* adj5 (innovation$ or intervention$ or technolog* or initiative*))).ti.  or ((("scaling" or widespread or spread or "rolling out" or "roll out" or "scale$ up" or "scale$ out" or upscaling or scalability or scalable or ((bring* or brought or taking or take* or increas* or going or implement* or econom*) adj5 scal*)) adj5 (innovation$ or intervention$ or technolog* or practice* or care or initiative* or program* or product* or therap* or service*)) or "reverse innovation*" or "trickle-up innovation*" or (transfer* adj5 (innovation$ or intervention$ or technolog* or initiative*))).ab.  or ((("scaling" or widespread or spread or "rolling out" or "roll out" or "scale$ up" or "scale$ out" or upscaling or scalability or scalable or ((bring* or brought or taking or take* or increas* or going or implement* or econom*) adj5 scal*)) adj5 (innovation$ or intervention$ or technolog* or practice* or care or initiative* or program* or product* or therap* or service*)) or "reverse innovation*" or "trickle-up innovation*" or (transfer* adj5 (innovation$ or intervention$ or technolog* or initiative*))).kw. | #5 | 26 575 |
| Scalability | #4 or #5 | #6 | 28 174 |
| Total result | #3 AND #6 | #7 | 2 585 |
| Filter for human | exp Animals/ NOT exp Humans/ | #8 | 4 558 766 |
| With filter for human | #7 NOT #8 | #9 | 2 528 |

### Embase-Elsevier (2019-03-20)

| **Concept** | **Keyword** | **Number** | **Result** |
| --- | --- | --- | --- |
| Tools for assessing (Controlled vocabulary) | 'questionnaire'/exp or 'survey'/exp or 'tool'/exp or 'score'/exp or 'rating scale'/exp or 'practice guideline'/exp or 'guideline'/exp or 'criterion variable'/exp or 'checklist'/exp or 'framework'/exp | #1 | 1 211 738 |
| Tools for assessing (Free text) | ((questionnaire* or survey* or instrument* or tool* or score* or scale* or guide* or criterion or checklist* or "check list*" or framework*) NEAR/4 (assess* or evaluat* or measur* or analy* or track* or observe or observing or observation* or predict*)):ti,ab,kw | #2 | 860 576 |
| Tools for assessing | #1 or #2 | #3 | 1 849 016 |
| Scalability (Controlled vocabulary) | 'scale up'/exp or 'scaling'/exp or 'scalability'/exp | #4 | 9 463 |
| Scalability (Free text) | (("scaling" or widespread or spread or "rolling out" or "roll out" or "scale up" or "scale out" or "scaled up" or "scaled out" or upscaling or scalability or scalable or "at scale") NEAR/5 (innovation* or intervention* or technolog* or practice* or care or initiative* or program* or product* or therap* or service*)):ti,ab,kw | #5 | 25 572 |
|  | ((scal* NEAR/5 ( brought or taking or take* or increas* or going or implement* or econom*)) and (innovation* or intervention* or technolog* or practice* or care or initiative* or program* or product* or therap* or service*)):ti,ab,kw | #6 | 11 927 |
|  | ("reverse innovation*" or "trickle-up innovation*"):ti,ab,kw | #7 | 42 |
|  | ((transfer*) NEAR/5 (innovation* or intervention* or technolog* or initiative*)):ti,ab,kw | #8 | 6 797 |
| Scalability (Free text) | #5 or #6 or #7 or #8 | #9 | 43 575 |
| Scalability | #4 or #9 | #10 | 50 616 |
| Total result | #3 AND #10 | #11 | 8 966 |
| Filter for human | 'animal'/exp not 'human'/exp | #12 | 5 209 908 |
| With filter for human | #11 NOT #12 | #13 | 8 815 |
| Embase | #13 AND [embase]/lim NOT ([embase]/lim AND [medline]/lim) | #14 | 3 453 |

### Web of Science (2019-03-20)

| **Concept** | **Keyword** | **Number** | **Result** |
| --- | --- | --- | --- |
| Tools for assessing (Free text) | TS=((questionnaire* or survey* or instrument* or tool* or score* or scale* or guide* or criterion or checklist* or "check list*" or framework*) NEAR/3 (assess* or evaluat* or measur* or analy* or track* or observe or observing or observation* or predict*)) | #1 | 1 193 464 |
| Scalability (Free text) | TS=((("scaling" or widespread or spread or "rolling out" or "roll out" or "scale up" or "scale out" or "scaled up" or "scaled out" or upscaling or scalability or scalable or "at scale" or ((bring* or brought or taking or take* or increas* or going or implement* or econom*) NEAR/4 scal*)) NEAR/4 (innovation* or intervention* or technolog* or practice* or care or initiative* or program* or product* or therap* or service*)) or "reverse innovation*" or "trickle-up innovation*" or (transfer* NEAR/4 (innovation* or intervention* or technolog* or initiative*))) | #2 | 74 977 |
| Health (Free text) | TS=(Health* or medicin* or medica* or clinic* or therap* or disease* or illness or diagnos* or treatment* or prevention* or drug* or remed* or pharmaceutic* or pill* or pharma* or healing* or surger* or infection* or patholog* or wound* or injur*) |  | 15 981 281 |
| Total result | #1 AND #2 | #3 | 1 800 |

### Cinahl-EBSCO (2019-03-20)

| **Concept** | **Keyword** | **Number** | **Result** |
| --- | --- | --- | --- |
| Tools for assessing (Controlled vocabulary) | (MH "Questionnaires+") or (MH "Surveys") or (MH "Research Instruments+") or (MH "Practice Guidelines") or (MH "Conceptual Framework") | #1 | 934 394 |
| Tools for assessing (Free text) | TI ((questionnaire# or survey# or instrument* or tool* or score* or scale# or guide* or criterion or checklist# or "check list*"or framework#) N3 (assess* or evaluat* or measur* or analy* or track* or observe or observing or observation* or predict*) ) OR AB ( (questionnaire# or survey# or instrument* or tool* or score* or scale# or guide* or criterion or checklist# or "check list*"or framework#) N3 (assess* or evaluat* or measur* or analy* or track* or observe or observing or observation* or predict*) ) OR SU ( (questionnaire# or survey# or instrument* or tool* or score* or scale# or guide* or criterion or checklist# or "check list*"or framework#) N3 (assess* or evaluat* or measur* or analy* or track* or observe or observing or observation* or predict*)) | #2 | 317 559 |
| Tools for assessing | #1 or #2 | #3 | 1 018 160 |
| Scalability (Controlled vocabulary) | (MH "Transferability") | #4 | 448 |
| Scalability (Free text) | TI ( (("scaling" or widespread or spread or "rolling out" or "roll out" or "scale up" or "scale out" or "scaled up" or "scaled out" or upscaling or scalability or scalable or ((bring* or brought or taking or take* or increas* or going or implement* or econom*) N4 scal*)) N4 (innovation# or intervention# or technolog* or practice* or care or initiative* or program* or product* or therap* or service*)) or "reverse innovation*" or "trickle-up innovation*" or (transfer* N4 (innovation# or intervention# or technolog* or initiative*)) ) OR AB ( (("scaling" or widespread or spread or "rolling out" or "roll out" or "scale up" or "scale out" or "scaled up" or "scaled out" or upscaling or scalability or scalable or ((bring* or brought or taking or take* or increas* or going or implement* or econom*) N4 scal*)) N4 (innovation# or intervention# or technolog* or practice* or care or initiative* or program* or product* or therap* or service*)) or "reverse innovation*" or "trickle-up innovation*" or (transfer* N4 (innovation# or intervention# or technolog* or initiative*)) ) OR SU ( (("scaling" or widespread or spread or "rolling out" or "roll out" or "scale up" or "scale out" or "scaled up" or "scaled out" or upscaling or scalability or scalable or ((bring* or brought or taking or take* or increas* or going or implement* or econom*) N4 scal*)) N4 (innovation# or intervention# or technolog* or practice* or care or initiative* or program* or product* or therap* or service*)) or "reverse innovation*" or "trickle-up innovation*" or (transfer* N4 (innovation# or intervention# or technolog* or initiative*)) ) | #5 | 6 989 |
| Scalability | #4 or #5 | #6 | 7 430 |
| Total result | #3 AND #6 | #7 | 2 361 |

### Eric-EBSCO (2019-03-20)

| **Concept** | **Keyword** | **Number** | **Result** |
| --- | --- | --- | --- |
| Tools for assessing (Controlled vocabulary) | DE "Questionnaires" OR DE "Surveys" OR DE "Community Surveys" OR DE "Mail Surveys" OR DE "National Surveys" OR DE "Occupational Surveys" OR DE "Online Surveys" OR DE "State Surveys" OR DE "Statistical Surveys" OR DE "Telephone Surveys" OR DE "Television Surveys" OR DE "Check Lists" OR DE "Measurement Instruments (1966 1980)" OR DE "Scaling" OR DE "Multidimensional Scaling" OR DE "Scores" OR DE "Cutting Scores" OR DE "Equated Scores" OR DE "Raw Scores" OR DE "True Scores" OR DE "Weighted Scores" OR DE "Rating Scales" OR DE "Behavior Rating Scales" OR DE "Likert Scales" OR DE "Semantic Differential" OR DE "Guidelines" OR DE "Facility Guidelines" OR DE "Guides" OR DE "Administrator Guides" OR DE "Laboratory Manuals" OR DE "Leaders Guides" OR DE "Program Guides OR DE "Criterion Referenced Tests" | #1 | 204 666 |
| Tools for assessing (Free text) | TI ( (questionnaire# or survey# or instrument* or tool* or score* or scale# or guide* or criterion or checklist# or "check list*"or framework#) N3 (assess* or evaluat* or measur* or analy* or track* or observe or observing or observation* or predict*) ) OR AB ( (questionnaire# or survey# or instrument* or tool* or score* or scale# or guide* or criterion or checklist# or "check list*"or framework#) N3 (assess* or evaluat* or measur* or analy* or track* or observe or observing or observation* or predict*) ) OR SU ( (questionnaire# or survey# or instrument* or tool* or score* or scale# or guide* or criterion or checklist# or "check list*"or framework#) N3 (assess* or evaluat* or measur* or analy* or track* or observe or observing or observation* or predict*) ) | #2 | 99 507 |
| Tools for assessing | S1 or S2 | #3 | 270 090 |
| Scalability (Controlled vocabulary) | Not available | - |  |
| Scalability (Free text) | TI ( (("scaling" or widespread or spread or "rolling out" or "roll out" or "scale up" or "scale out" or "scaled up" or "scaled out" or upscaling or scalability or scalable or ((bring* or brought or taking or take* or increas* or going or implement* or econom*) N4 (scal*))) N4 (innovation# or intervention# or technolog* or practice* or care or initiative* or program* or product* or therap* or service*)) or "reverse innovation*" or "trickle-up innovation*" or ((transfer*) N4 (innovation# or intervention# or technolog* or initiative*)) ) OR AB ( (("scaling" or widespread or spread or "rolling out" or "roll out" or "scale up" or "scale out" or "scaled up" or "scaled out" or upscaling or scalability or scalable or ((bring* or brought or taking or take* or increas* or going or implement* or econom*) N4 (scal*))) N4 (innovation# or intervention# or technolog* or practice* or care or initiative* or program* or product* or therap* or service*)) or "reverse innovation*" or "trickle-up innovation*" or ((transfer*) N4 (innovation# or intervention# or technolog* or initiative*)) ) OR SU ( (("scaling" or widespread or spread or "rolling out" or "roll out" or "scale up" or "scale out" or "scaled up" or "scaled out" or upscaling or scalability or scalable or ((bring* or brought or taking or take* or increas* or going or implement* or econom*) N4 (scal*))) N4 (innovation# or intervention# or technolog* or practice* or care or initiative* or program* or product* or therap* or service*)) or "reverse innovation*" or "trickle-up innovation*" or ((transfer*) N4 (innovation# or intervention# or technolog* or initiative*)) ) | #4 | 4 741 |
| Health (Controlled vocabulary) | DE "Health" OR DE "Child Health" OR DE "Mental Health" OR DE "Occupational Safety and Health" OR DE "Physical Health" OR DE "Public Health" OR DE "Wellness" OR DE "Child Health" OR DE "Mental Health" OR DE "Occupational Safety and Health" OR DE "Physical Health" OR DE "Dental Health" OR DE "Access to Health Care" OR DE "Health Care Costs" OR DE "Allied Health Occupations" OR DE "Health Personnel" OR DE "Mental Health Workers" OR DE "Nurses" OR DE "Physicians" OR DE "Psychologists" OR DE "Allied Health Personnel" OR DE "Emergency Medical Technicians" OR DE "Home Health Aides" OR DE "Mental Health Workers" OR DE "School Psychologists" OR DE "School Nurses" OR DE "Foreign Medical Graduates" OR DE "Health Services" | #5 | 54 600 |
|  | DE "Community Health Services" OR DE "Hospices (Terminal Care)" OR DE "Medical Services" OR DE "Prenatal Care" OR DE "School Health Services" OR DE "Prenatal Care" OR DE "School Health Services" OR DE "Health Activities" OR DE "Health Promotion" OR DE "Health Behavior" OR DE "Health Care Costs" | #6 | 20 902 |
|  | DE "Medical Care Evaluation" OR DE "Health Conditions" OR DE "Health Facilities" OR DE "Nursing Homes" OR DE "Health Materials" OR DE "Health Needs" OR DE "Health Sciences" OR DE "Health Programs" OR DE "Immunization Programs" OR DE "Mental Health Programs OR DE "Medicine" OR DE "Anesthesiology" OR DE "Audiology" OR DE "Biomedicine" OR DE "Dentistry" OR DE "Dietetics" OR DE "Epidemiology" | #7 | 9 011 |
|  | DE "Family Practice (Medicine)" OR DE "Geriatrics" OR DE "Gynecology" OR DE "Internal Medicine" OR DE "Neurology" OR DE "Nursing" OR DE "Obstetrics" OR DE "Oncology"OR DE "Ophthalmology" OR DE "Pathology" OR DE "Pediatrics" OR DE "Pharmacology" OR DE "Pharmacy" OR DE "Podiatry" OR DE "Preventive Medicine" OR DE "Primary Health Care" OR DE "Psychiatry" OR DE "Sports Medicine" OR DE "Surgery" | #8 | 14 681 |
|  | DE "Toxicology" OR DE "Psychopathology" OR DE "Speech Language Pathology" OR DE "Clinical Experience" OR DE "Medical Research" OR DE "Clinical Diagnosis" OR DE "Medical Evaluation" OR DE "Auditory Evaluation" OR DE "Dental Evaluation" OR DE "Physical Examinations" OR DE "Speech Evaluation" OR DE "Clinics" OR DE "Psychoeducational Clinics" OR DE "Hearing (Physiology)" OR DE "Therapy" OR DE "Art Therapy" OR DE "Bibliotherapy" OR DE "Drug Therapy" OR DE "Educational Therapy" OR DE "Group Therapy" OR DE "Hearing Therapy" OR DE "Music Therapy" | #9 | 31 358 |
|  | DE "Occupational Therapy" OR DE "Physical Therapy" OR DE "Psychotherapy" OR DE "Speech Therapy" OR DE "Therapeutic Recreation" OR DE "Diseases" OR DE "Alcoholism" OR DE "Allergy" OR DE "Alzheimers Disease" OR DE "Cancer" OR DE "Chronic Illness" OR DE "Communicable Diseases" OR DE "Diabetes" OR DE "Drug Addiction" OR DE "Eating Disorders" OR DE "Fetal Alcohol Syndrome" OR DE "Genetic Disorders" OR DE "Hypertension" OR DE "Obesity" OR DE "Occupational Diseases" OR DE "Poisoning" OR DE "Seizures" OR DE "Terminal Illness" OR DE "Communicable Diseases" OR DE "Acquired Immunodeficiency Syndrome (AIDS)" OR DE "Rubella" OR DE "Sexually Transmitted Diseases" OR DE "Injuries" OR DE "Head Injuries" | #10 | 31 095 |
|  | #5 or #6 or #7 or #8 or #9 or #10 | #11 | 117 677 |
| Health (Free text) | TI ( health* or medicin* or medica* or clinic* or therap* or disease* or illness or diagnos* or treatment* or prevention* or drug* or remed* or pharmaceutic* or pill# or pharma* or healing* or surger* or infection* or patholog* or wound* or injur* ) OR AB ( health* or medicin* or medica* or clinic* or therap* or disease* or illness or diagnos* or treatment* or prevention* or drug* or remed* or pharmaceutic* or pill# or pharma* or healing* or surger* or infection* or patholog* or wound* or injur* ) OR SU ( health* or medicin* or medica* or clinic* or therap* or disease* or illness or diagnos* or treatment* or prevention* or drug* or remed* or pharmaceutic* or pill# or pharma* or healing* or surger* or infection* or patholog* or wound* or injur* ) | #12 | 269 267 |
| Health | S5 OR S6 | #13 | 282 569 |
| Total result | S3 AND S4 AND S13 | #14 | 147 |

### Cochrane Library (2019-03-20)

| **Concept** | **Keyword** | **Number** | **Result** |
| --- | --- | --- | --- |
| Tools for assessing (Controlled vocabulary) | MeSH descriptor: [Surveys and Questionnaires] explode all trees | #1 | 50 610 |
|  | MeSH descriptor: [Guideline] explode all trees | #2 | 0 |
|  | MeSH descriptor: [Checklist] explode all trees | #3 | 233 |
|  | #1 or #2 or #3 | #4 | 50 781 |
| Tools for assessing (Free text) | ((questionnaire* or survey* or instrument* or tool* or score* or scale* or guide* or criterion or checklist* or "check list*" or framework*) NEAR/3 (assess* or evaluat* or measur* or analy* or track* or observe or observing or observation* or predict*)):ti,ab,kw | #5 | 63 980 |
| Tools for assessing | #4 or #5 | #6 | 106 620 |
| Scalability (Controlled vocabulary) | Not available | NA | - |
| Scalability (Free text) | (("scaling" or widespread or spread or "rolling out" or "roll out" or "scale up" or "scale out" or "scaled up" or "scaled out" or upscaling or scalability or scalable) NEAR/4 (innovation* or intervention* or technolog* or practice* or care or initiative* or program* or product* or therap* or service*)):ti,ab,kw | #7 | 1 353 |
|  | (((bring* or brought or taking or take* or increas* or going or implement* or econom*) NEAR/4 (scal*)) NEAR/4 (innovation* or intervention* or technolog* or practice* or care or initiative* or program* or product* or therap* or service*)):ti,ab,kw | #8 | 160 |
|  | ("reverse innovation*" or "trickle-up innovation*"):ti,ab,kw | #9 | 2 |
|  | ((transfer*) NEAR/4 (innovation* or intervention* or technolog* or initiative*)):ti,ab,kw | #10 | 292 |
| Scalability | #7 or #8 or #9 or #10 | #11 | 1770 |
| Total result | #6 AND #11 | #12 | 337 |

### PsycInfo-Ovid (2019-03-20)

| **Concept** | **Keyword** | **Number** | **Result** |
| --- | --- | --- | --- |
| Tools for assessing (Controlled vocabulary) | exp questionnaires/ or surveys/ or mail surveys/ or telephone surveys/ or score equating/ or cutting scores/ or standard scores/ or "scoring (testing)"/ or exp "scaling (testing)"/ or exp criterion referenced tests/ or "checklist (testing)"/ | #1 | 36 178 |
| Tools for assessing (Free text) | ((questionnaire$ or survey$ or instrument* or tool* or score* or scale$ or guide* or criterion or checklist$ or "check list$" or framework$) adj4 (assess* or evaluat* or measur* or analy* or track* or observe or observing or observation* or predict*)).ti.  or ((questionnaire$ or survey$ or instrument* or tool* or score* or scale$ or guide* or criterion or checklist$ or framework$) adj4 (assess* or evaluat* or measur* or analy* or track* or observe or observing or observation* or predict*)).ab.  or ((questionnaire$ or survey$ or instrument* or tool* or score* or scale$ or guide* or criterion or checklist$ or framework$) adj4 (assess* or evaluat* or measur* or analy* or track* or observe or observing or observation* or predict*)).kw. | #2 | 293 375 |
| Tools for assessing | #1 or #2 | #3 | 316 309 |
| Scalability (Controlled vocabulary) | exp technology transfer/ | #4 | 193 |
| Scalability (Free text) | ((("scaling" or widespread or spread or "rolling out" or "roll out" or "scale$ up" or "scale$ out" or upscaling or scalability or scalable or ((bring* or brought or taking or take* or increas* or going or implement* or econom*) adj5 scal*)) adj5 (innovation$ or intervention$ or technolog* or practice* or care or initiative* or program* or product* or therap* or service*)) or "reverse innovation*" or "trickle-up innovation*" or (transfer* adj5 (innovation$ or intervention$ or technolog* or initiative*))).ti.  or ((("scaling" or widespread or spread or "rolling out" or "roll out" or "scale$ up" or "scale$ out" or upscaling or scalability or scalable or ((bring* or brought or taking or take* or increas* or going or implement* or econom*) adj5 scal*)) adj5 (innovation$ or intervention$ or technolog* or practice* or care or initiative* or program* or product* or therap* or service*)) or "reverse innovation*" or "trickle-up innovation*" or (transfer* adj5 (innovation$ or intervention$ or technolog* or initiative*))).ab.  or ((("scaling" or widespread or spread or "rolling out" or "roll out" or "scale$ up" or "scale$ out" or upscaling or scalability or scalable or ((bring* or brought or taking or take* or increas* or going or implement* or econom*) adj5 scal*)) adj5 (innovation$ or intervention$ or technolog* or practice* or care or initiative* or program* or product* or therap* or service*)) or "reverse innovation*" or "trickle-up innovation*" or (transfer* adj5 (innovation$ or intervention$ or technolog* or initiative*))).kw. | #5 | 6 678 |
| Scalability | #4 or #5 | #6 | 6 678 |
| Total result | #3 AND #6 | #7 | 673 |
