# Supplementary material for: Tools for assessing the scalability of innovations in health: a systematic review
Source: Health Res Policy Syst. 2022 Mar 24;20:34. doi: 10.1186/s12961-022-00830-5 (PMC8943495; doi:10.1186/s12961-022-00830-5)
Supplement: Supplementary file 2 — Additional file 2. List of relevant websites used to identify potential eligible records. [file 12961_2022_830_MOESM2_ESM.docx]

**Additional file 2:** List of relevant websites used to identify potential eligible records

| **Name of organization** | **Website** | **Date** |
| --- | --- | --- |
| Canadian Foundation for Healthcare Improvement (CFHI) | <https://www.cfhi-fcass.ca/> | December 20, 2019 |
| Institut National d'Excellence en Santé et Services Sociaux (INESSS) | <https://www.inesss.qc.ca/> | December 20, 2019 |
| National Institutes of Health (NIH) | <https://www.nih.gov/> | December 20, 2019 |
| Institute for Healthcare Improvement (IHI) | [www.ihi.org](http://www.ihi.org) | December 20, 2019 |
| United States Agency for International Development (USAID) | https://www.usaid.gov/ | December 20, 2019 |
| National Implementation Research Network | https://nirn.fpg.unc.edu/national-implementation-research-network | December 20, 2019 |
| The State Implementation & Scaling‐up of Evidence‐based Practices Center (SISEP) | <https://sisep.fpg.unc.edu/> | December 20, 2019 |
| Australian Prevention Partnership Centre | <https://preventioncentre.org.au/> | December 19, 2019 |
| Agency for Healthcare Research and Quality (AHRQ) | <https://www.ahrq.gov/> | December 19, 2019 |
| European Implementation Collaborative | <https://www.implementation.eu/> | December 6, 2019 |
| WHO/ExpandNet: | <http://expandnet.net/biblio/> | November 21, 2019 |
| Global Reporting Initiative | <https://www.globalreporting.org/Pages/default.aspx> | November 21, 2019 |
| The Health Foundation | <https://www.health.org.uk/> | November 21, 2019 |
| Google search (+Google scholar) [examining the first 200 hits] | [www.google.com](http://www.google.com) (<https://scholar.google.com/>) | October 31, 2019 |
| What Works Clearinghouse | <https://ies.ed.gov/ncee/wwc/> | October 30, 2019 |
| Bill & Melinda Gates Foundation | <https://www.gatesfoundation.org/> | October 29, 2019 |
| Scaling Pathways | <http://scalingpathways.globalinnovationexchange.org/> | October 29, 2019 |
| UNICEF | <https://www.unicef.org/> | October 29, 2019 |
| Comet register | <http://www.comet-initiative.org/> | October 25, 2019 |
| The World Bank | <http://www.worldbank.org/> | October 25, 2019 |
| The Wallace Foundation | <https://www.wallacefoundation.org/> | October 25, 2019 |
| Dissemination and Implementation Models | <http://dissemination-implementation.org/index.aspx> | October 24, 2019 |
| AcademyHealth | <https://www.academyhealth.org/> | October 23, 2019 |
| The Evidence Project | <http://evidenceproject.popcouncil.org/> | October 23, 2019 |
| NSW Government (Centre for Epidemiology and Evidence NSW Ministry of Health) | <https://www.health.nsw.gov.au/Pages/default.aspx> | October 10, 2019 |
| International Development Research Centre | <https://www.idrc.ca/en> [<https://idl-bnc-idrc.dspacedirect.org/>] | October 9, 2019 |

**Keywords used**

- **In English:** scalability, transferability, readiness, scale, scaling, upscaling, up-scaling, spread
- **In French:** Potentiel de mise à l’échelle, potentiel de passage à grande échelle, transférabilité, mise à l'échelle, passage à grande échelle, accroissement d'échelle, passage à l'échelle, diffusion
