# Supplementary material for: Tools for assessing the scalability of innovations in health: a systematic review
Source: Health Res Policy Syst. 2022 Mar 24;20:34. doi: 10.1186/s12961-022-00830-5 (PMC8943495; doi:10.1186/s12961-022-00830-5)
Supplement: Supplementary file 3 — Additional file 3. Email sent to experts to identify potential eligible records. [file 12961_2022_830_MOESM3_ESM.docx]

**Additional file 3:** Email sent to experts to identify potential eligible records

| **Subject** | Request for information regarding your project on scalability |
| --- | --- |
| **Text** | Dear Colleague,  My name is [Ali Ben Charif](https://nam10.safelinks.protection.outlook.com/?url=http%3A%2F%2Fwww.decision.chaire.fmed.ulaval.ca%2Fequipe-en%2F5b63a96d5b489b0334183459&data=02%7C01%7C%7Cd18fd552b6724cb1953008d7ee41447f%7C84df9e7fe9f640afb435aaaaaaaaaaaa%7C1%7C0%7C637239836393621006&sdata=7y0qTOqZKroM9LKGMSCxIbdAnwtvDobAuQvX7T3lnF0%3D&reserved=0) and I am a postdoctoral researcher in Implementation Science with [Dr. France Légaré](https://nam10.safelinks.protection.outlook.com/?url=http%3A%2F%2Fwww.decision.chaire.fmed.ulaval.ca%2Ffrance-legare-en&data=02%7C01%7C%7Cd18fd552b6724cb1953008d7ee41447f%7C84df9e7fe9f640afb435aaaaaaaaaaaa%7C1%7C0%7C637239836393621006&sdata=UgU6hQa5oKiJQ5exii4MwLkG2L%2BviVmNorX07%2FcUe1g%3D&reserved=0) at Laval University in Canada.  We have identified you as a stakeholder in the science and practice of scaling up in health and would appreciate if you could share documentation on any tools you may have developed for assessing the **scalability** of health initiatives.  We are currently conducting a systematic review (entitled "[Tools for assessing the scalability of health innovations: a systematic review](https://nam10.safelinks.protection.outlook.com/?url=https%3A%2F%2Fwww.crd.york.ac.uk%2Fprospero%2Fdisplay_record.php%3FID%3DCRD42019107095&data=02%7C01%7C%7Cd18fd552b6724cb1953008d7ee41447f%7C84df9e7fe9f640afb435aaaaaaaaaaaa%7C1%7C0%7C637239836393631001&sdata=0YvBh6T7%2F%2FWTKykcSAf4Zkfquu74O8nrcrWn4qwPJfc%3D&reserved=0)") aiming to review tools (e.g., criteria, checklists, questionnaires, frameworks) that have been developed for assessing the potential for health initiatives to be scaled up. We would like to make sure the work you have produced on this area is included in our review.  We would appreciate if you could let us know by **May 29, 2020**. Please do not hesitate to contact us for further details.  Thank you very much for your help.  Best regards,  Ali  ---  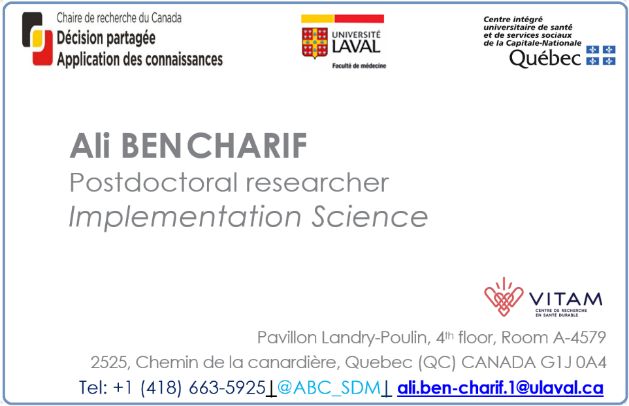 |
