# Supplementary material for: Tools for assessing the scalability of innovations in health: a systematic review
Source: Health Res Policy Syst. 2022 Mar 24;20:34. doi: 10.1186/s12961-022-00830-5 (PMC8943495; doi:10.1186/s12961-022-00830-5)
Supplement: Supplementary file 4 — Additional file 4. Records selection process. [file 12961_2022_830_MOESM4_ESM.docx]

**Additional file 4:** Records selection process

- 1. **Records assignment (n=8,494)**

| **Reviewer** | | **Pilot 1 (n=425)** | **Pilot 2 (n=406)** | **Other records screened (n=7,663)** | | | |
| --- | --- | --- | --- | --- | --- | --- | --- |
|  |  | **(5%, 425/8494)** | **(5%, 406/8069)** | n=3497 | n=1500 | n=1500 | n=1163 |
| **Reviewer 1** | **MAS** | ☑ | ☑ | ☑ | ☑ | ☑ | ☑ |
| **Reviewer 2** | **ABC** | ☑ | ☑ | ☑ |  |  |  |
|  | **AG** | ☑ | ☑ |  | ☑ |  |  |
|  | **JM** | ☑ | ☑ |  |  | ☑ |  |
|  | **YO** | ☑ | ☑ |  |  |  | ☑ |

- 1. **Weighted Cohen’s kappa calculated with pilot 2 (n=406)**

| **Reviewer** | **MAS** | **ABC** | **AG** | **JM** |
| --- | --- | --- | --- | --- |
| **ABC** | 86% |  |  |  |
| **AG** | 75% | 86% |  |  |
| **JM** | 75% | 89% | 80% |  |
| **YO** | 66% | 89% | 75% | 75% |
