# Supplementary material for: Tools for assessing the scalability of innovations in health: a systematic review
Source: Health Res Policy Syst. 2022 Mar 24;20:34. doi: 10.1186/s12961-022-00830-5 (PMC8943495; doi:10.1186/s12961-022-00830-5)
Supplement: Supplementary file 5 — Additional file 5. List of excluded reports with reason for exclusion. [file 12961_2022_830_MOESM5_ESM.docx]

**Additional file 5:** List of excluded reports with reason for exclusion

| **Authors [Reference]** | **Year of publication** | **Reason for exclusion** |
| --- | --- | --- |
| Aamir et al. [81] | 2018 | The tool is not intended to be used for assessing the scalability of an innovation |
| Agbakoba et al. [85] | 2016 | The tool is not intended to be used for assessing the scalability of an innovation |
| Bacci et al. [104] | 2016 | The study did not present or describe the development or validation of a tool |
| Brink et al. [80] | 1995 | The study did not present or describe the development or validation of a tool |
| Carlfjord et al. [88] | 2010 | The study did not present or describe the development or validation of a tool |
| Carr et al. [77] | 2014 | The study did not present or describe the development or validation of a tool |
| Davies et al. [65] | 2016 | The study did not present or describe the development or validation of a tool |
| Diwan [79] | 1994 | The study did not present or describe the development or validation of a tool |
| Egeland et al. [97] | 2016 | The study did not present or describe the development or validation of a tool |
| Ezeanochie [83] | 2018 | The study did not present or describe the development or validation of a tool |
| Fischer et al. [95] | 2015 | The study did not present or describe the development or validation of a tool |
| Flynn et al. [68] | 2012 | The study did not present or describe the development or validation of a tool |
| Fogerty et al. [94] | 2016 | The tool is not intended to be used for assessing the scalability of an innovation |
| Forkuo-Minka A [90] | 2018 | The tool is not intended to be used for assessing the scalability of an innovation |
| Goeree et al. [109] | 2011 | The study did not present or describe the development or validation of a tool |
| Grooten et al. [92] | 2017 | Wrong type of document |
| Grooten et al. [71] | 2019 | The tool is not intended to be used for assessing the scalability of an innovation |
| Gyldmark et al. [87] | 2018 | The study did not present or describe the development or validation of a tool |
| Huang et al. [66] | 2011 | The study did not present or describe the development or validation of a tool |
| Kastner et al.[105] | 2017 | The study did not present or describe the development or validation of a tool |
| Kliche et al. [89] | 2012 | The study did not present or describe the development or validation of a tool |
| Leon et al. [70] | 2012 | The tool is not intended to be used for assessing the scalability of an innovation |
| Maar et al. [84] | 2015 | The tool is not intended to be used for assessing the scalability of an innovation |
| Marshall et al. [114] | 2014 | The tool is not intended to be used for assessing the scalability of an innovation |
| Morgenthaler et al. [115] | 2012 | The tool is not intended to be used for assessing the scalability of an innovation |
| Nguyen [99] | 2017 | The tool is not intended to be used for assessing the scalability of an innovation |
| Nguyen et al. [78] | 2017 | The tool is not intended to be used for assessing the scalability of an innovation |
| O'Hara et al. [112] | 2014 | The tool is not intended to be used for assessing the scalability of an innovation |
| Pednekar et al. [102] | 2018 | The study did not present or describe the development or validation of a tool |
| Perez-Escamilla et al. [73] | 2017 | The tool is not intended to be used for assessing the scalability of an innovation |
| Perla et al. [91] | 2013 | The tool is not intended to be used for assessing the scalability of an innovation |
| Renju et al. [64] | 2010 | The study did not present or describe the development or validation of a tool |
| Renju et al. [101] | 2010 | The study did not present or describe the development or validation of a tool |
| Rohrbach et al. [113] | 2006 | The tool is not intended to be used for assessing the scalability of an innovation |
| Rosas et al. [75] | 2016 | The tool is not intended to be used for assessing the scalability of an innovation |
| Rosella et al. [106] | 2016 | The tool is not intended to be used for assessing the scalability of an innovation |
| Sako et al. [100] | 2017 | The study did not present or describe the development or validation of a tool |
| Sako et al. [82] | 2018 | The tool is not intended to be used for assessing the scalability of an innovation |
| Sani et Arshad [108] | 2015 | The tool is not intended to be used for an innovation in the field of health |
| Schloemer et Schroder-Back [76] | 2017 | Wrong type of document |
| Shamu et al. [96] | 2016 | The study did not present or describe the development or validation of a tool |
| Simpson [61] | 2002 | The tool is not intended to be used for assessing the scalability of an innovation |
| Southon et al. [86] | 1997 | The study did not present or describe the development or validation of a tool |
| Spicer et al. [60] | 2016 | The tool is not intended to be used for assessing the scalability of an innovation |
| Spicer et al. [59] | 2018 | The study did not present or describe the development or validation of a tool |
| Upton et al. [107] | 2014 | The tool is not intended to be used for assessing the scalability of an innovation |
| Vanderkruik et McPherson [62] | 2017 | The study did not present or describe the development or validation of a tool |
| Werfel et al. [103] | 2017 | The study did not present or describe the development or validation of a tool |
| Essers et al. [110] | 2010 | The tool is not intended to be used for assessing the scalability of an innovation |
| Grooten et al. [69] | 2018 | The tool is not intended to be used for assessing the scalability of an innovation |
| Hill et al. [98] | 2019 | The tool is not intended to be used for assessing the scalability of an innovation |
| KalÓ et al. [111] | 2012 | The tool is not intended to be used for assessing the scalability of an innovation |
| Madden et al. [116] | 2017 | The tool is not intended to be used for assessing the scalability of an innovation |
| Miller [117] | 2010 | Wrong type of document |
| Pérez‐Escamilla et al. [74] | 2018 | The tool is not intended to be used for assessing the scalability of an innovation |
| Berger et al. [72] | 2009 | The tool is not intended to be used for assessing the scalability of an innovation |
| Houck et al. [63] | 2009 | The tool is not intended to be used for assessing the scalability of an innovation |
| Joly et al. [93] | 2012 | The tool is not intended to be used for assessing the scalability of an innovation |
| Toobert et al. [67] | 2012 | The study did not present or describe the development or validation of a tool |
