# Supplementary material for: Tools for assessing the scalability of innovations in health: a systematic review
Source: Health Res Policy Syst. 2022 Mar 24;20:34. doi: 10.1186/s12961-022-00830-5 (PMC8943495; doi:10.1186/s12961-022-00830-5)
Supplement: Supplementary file 7 — Additional file 7. Interpretability criteria for selecting items. [file 12961_2022_830_MOESM7_ESM.docx]

**Additional file 7:** Interpretability criteria for selecting items

| **#** | **Criterion** | **Description** | **Response** | | |
| --- | --- | --- | --- | --- | --- |
|  |  |  | **Met** | **Unmet** | **Unclear** |
| 1 | The item captures the concept that is intended | The content of the item must be able to assess the concepts of the study (i.e., scalability). | ○ | ○ | ○ |
| 2 | The item is relevant to all innovations of the target setting | The content of the item must be relevant to all innovations of the target setting (e.g., primary health care, public health). | ○ | ○ | ○ |
| 3 | The item is worded in a manner consistent with expressions used by end-users | The vocabularies used in the formulation of the item must be easily understood by end-users (e.g., patients) or must not be technical. | ○ | ○ | ○ |
| 4 | The item is comprehensible, i.e. not ambiguous or poorly worded | The wording of the item must be clear and unambiguous for its interpretation. | ○ | ○ | ○ |
| 5 | The item represents a single concept, rather than a multidimensional concept | The formulation of the item must be done with a single concept, instead of a multidimensional concept. | ○ | ○ | ○ |
| 6 | The item does not contain the words ‘and’, ‘or’, or ‘because’ | Avoid the use of “and”, “or”, or “because” in the wording of the item. | ○ | ○ | ○ |
| 7 | The item is not likely to be vulnerable to ceiling or floor effects within the target innovations, i.e., it will change with innovation | The content of the item must be over time, it should not be vulnerable to ceiling or floor effects within the target innovations.  Ceiling or floor effects occur when the tests or scales are relatively easy or difficult such that substantial proportions of individuals (i.e., innovations) obtain either maximum or minimum scores and that the true extent of their abilities cannot be determined. | ○ | ○ | ○ |
| 8 | The content of the item is appropriate for the recall period | The content of the item must be appropriate to the period of time that respondents are asked to consider in responding to the item question, including event-driven immediate, daily, up to weekly, and longer than weekly recall periods. | ○ | ○ | ○ |
| 9 | The content of the item is appropriate for the mode of administration | The content of the item must be appropriate to the method of data collection (i.e., self-administered or interviewer-administered questionnaires). | ○ | ○ | ○ |
| 10 | The item is as short as possible, although not so short that comprehensibility is lost | The item should be as short as possible whilst maintaining comprehensibility to end-users. | ○ | ○ | ○ |
| 11 | The item does not contain negated constructs or negative answers | The item should not be constructed with sentences in which there is a negation (e.g., no control, no adaptation) that promotes a negative response (e.g., never). | ○ | ○ | ○ |
| 12 | The item does not ask a combination of two or more questions | Avoid items with the combination of two or more questions. | ○ | ○ | ○ |
| 13 | The item does not ask excessively personal questions | The item must not have exclusively personal or intrusive content which may lead to missing values ​​or annoy the respondents. | ○ | ○ | ○ |
| 14 | The item is ethically appropriate | It must be ensured that the content of the item is appropriate and does not harm to beliefs about what is morally right and wrong for all respondents including potentially vulnerable subgroups. | ○ | ○ | ○ |
| 15 | The item does not refer to circumstances, situations or lifestyles that may not be universal across all responders | The item should be avoided whose content is not appropriate for all participants. | ○ | ○ | ○ |
